# Native aggregation is a common feature among triosephosphate isomerases of different species

**DOI:** 10.1038/s41598-020-58272-4

**Published:** 2020-01-28

**Authors:** Mónica Rodríguez-Bolaños, Héctor Miranda-Astudillo, Edgar Pérez-Castañeda, Diego González-Halphen, Ruy Perez-Montfort

**Affiliations:** 1 Departamento de Bioquímica y Biología Estructural, Instituto de Fisiología Celular, Universidad Nacional Autónoma de México, Ciudad de Mexico, Mexico; 2 Departamento de Genética Molecular, Instituto de Fisiología Celular, Universidad Nacional Autónoma de México, Ciudad de Mexico, Mexico

**Keywords:** Proteins, Biochemistry, Chemical biology

## Abstract

Triosephosphate isomerase (TIM) is an enzyme of the glycolysis pathway which exists in almost all types of cells. Its structure is the prototype of a motif called TIM-barrel or (α/β)_8_ barrel, which is the most common fold of all known enzyme structures. The simplest form in which TIM is catalytically active is a homodimer, in many species of bacteria and eukaryotes, or a homotetramer in some archaea. Here we show that the purified homodimeric TIMs from nine different species of eukaryotes and one of an extremophile bacterium spontaneously form higher order aggregates that can range from 3 to 21 dimers per macromolecular complex. We analysed these aggregates with clear native electrophoresis with normal and inverse polarity, blue native polyacrylamide gel electrophoresis, liquid chromatography, dynamic light scattering, thermal shift assay and transmission electron and fluorescence microscopies, we also performed bioinformatic analysis of the sequences of all enzymes to identify and predict regions that are prone to aggregation. Additionally, the capacity of TIM from *Trypanosoma brucei* to form fibrillar aggregates was characterized. Our results indicate that all the TIMs we studied are capable of forming oligomers of different sizes. This is significant because aggregation of TIM may be important in some of its non-catalytic moonlighting functions, like being a potent food allergen, or in its role associated with Alzheimer’s disease.

## Introduction

Protein concentration reaches 80–400 mg/mL in intracellular space^1,2^ so interaction between multiple proteins is unavoidable and must be a very controlled process. Native aggregation can be the ordered interaction between proteins in response to muscle contraction, hypoxia, increased secretion activity, increase in temperature, mechanical stress, change in hydrostatic pressure, application of an electrical current, formation of an action potential, variation of pH, variation in the tonicity (osmolarity) of the medium, or the presence of heavy metal salts^3^. One of the notorious evidences of native aggregation is the change in viscosity and turbidity of the cytoplasm under non-pathological conditions; these changes are temporary and reversible so that cell function can be corrected as needed^4^.

Native aggregation usually begins as a response to a cellular demand and can involve: formation of binding sites to ions, molecules or solutes, an increase in enzyme activity, formation of channels producing intercellular contacts, etc.^5,6^. Most proteins have low propensities to aggregate, because its hydrophobic amino acids are usually hidden from the solvent, but changes in solvent composition, heat, or pressure, reduce the thermodynamic stability of these proteins by promoting the exposure of some residues or hydrophobic surfaces^7^. Thus, under certain circumstances, peptides rearrange to adapt to changes, initiating the process of reversible or irreversible aggregation, as in certain pathological conditions. For example, when the hydrophobic residues of a protein are exposed, these can interact with other proteins in similar conditions, producing their co-aggregation^8^. *In vitro*, the reversibility or irreversibility of aggregation depends on factors like solvent composition, pH, temperature and time^9^. The aggregates can be of various types: some are small (dimeric, trimeric, tetrameric) and reversible; others are irreversible with non-covalent interactions, and yet others are covalent oligomers (joined by disulphide bridges). Some are large aggregates (of over 10 units) that may, or may not, be reversible, and there are also very large aggregates whose size can vary from 50 nm to 3 µm^9^.

Triosephosphate isomerase (TIM) is an enzyme of the glycolysis pathway that catalyses the isomerization of glyceraldehyde 3-phosphate (GAP) to dihydroxyacetone phosphate (DHAP). TIM is present in almost all organisms^10^, it has approximately 250 amino acids per monomer (27000 Da), although its length may vary depending on the species. The structure of TIM is that of an (α/β)_8_ barrel, also known as TIM barrel^11^. This is the most frequent structural motif in proteins (10% of the known enzyme structures exhibit this motif)^12^.

Due to an apparent increase in amyloid diseases, studying the mechanisms of protein aggregation is of interest. TIM forms irreversible fibrillary-type aggregates in pathological conditions and it is one of the proteins associated with Alzheimer’s disease, a pathological condition in which high oxidative stress causes the modification of several proteins. TIM is one of the main proteins that undergoes nitrotyrosination, a modification that promotes its aggregation with the amyloid beta-peptide (Abeta)^13,14^, a largely unstructured and flexible peptide, mainly related with aberrant aggregation, whose physiological function in healthy individuals remains largely unclear. Nevertheless, it has been proposed to be involved in learning and memory processes^15,16^. Also, the formation of rabbit TIM oligomeric aggregates of 1320 kDa has been evidenced *in vitro* using blue native gels^17^. Here we extended these findings, and identified different oligomeric states for TIMs purified from ten different species, using a combination of techniques.

## Results and Discussion

### Expression and purification of TIMs from ten different species

**Figure 1:**
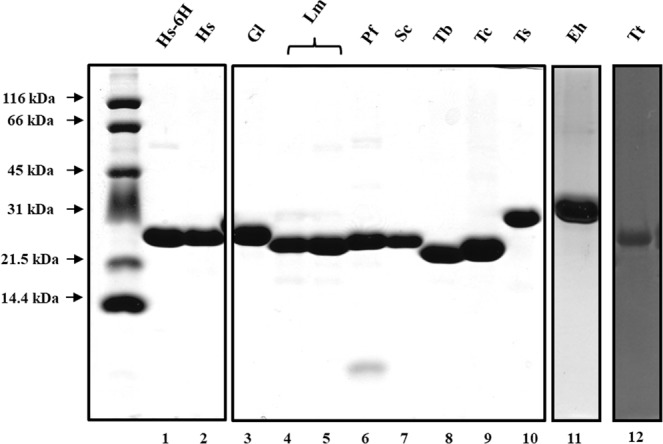
SDS-PAGE of purified TIMs from all species used in this work. Tricine-SDS polyacrylamide gel showing purified TIMs from: Gl: *Giardia lamblia* (GlTIM), Sc: *Saccharomyces cerevisiae* (ScTIM), Pf: *Plasmodium falciparum* (PfTIM), Eh: *Entamoeba histolytica* (EhTIM), Lm: *Leishmania mexicana* (LmTIM), Hs-6H: *Homo sapiens* with histidine tag (HsTIMHIS), Hs: *Homo sapiens* (HsTIM), Ts: *Taenia solium* (TsTIM), Tt: *Thermus thermophilus* (TtTIM), Tc: *Trypanosoma cruzi* (TcTIM), Tb: *Trypanosoma brucei* (TbTIM). The molecular mass of the markers is indicated on the left side.

In order to characterize the oligomeric profile of the native aggregates of this enzyme a highly purified protein is required. The heterologous expression and purification of the TIMs from ten different species was carried out as mentioned in material and methods (sections 3.1, 3.2 and 3.3) Fig. 1 shows the SDS-PAGE electrophoretic pattern of the purified enzymes. All TIMs showed a single polypeptide band and only an additional low molecular mass band could be observed in the sample of *Plasmodium falciparum* TIM (PfTIM) (Fig. 1, lane 6). As shown in Table 1 the specific activity and the kinetic parameters for nine TIMs are in the range reported previously for each of them^18–24^. In the case of *Thermus thermophilus* TIM (TtTIM), to our knowledge, no *in vitro* activity has been reported previously. The activity of PfTIM is the lowest for all the TIMs studied, but the Kcat/km value is similar to the ones determined for the rest of the enzymes (Table 1).

**Table 1:** Specific activity, kinetic and physico-chemical parameters of TIMs from several species and physico-chemical parameters of other oligomer-forming proteins.

Organism or protein	Specific activity* (µmol/min/mg protein)	Km* (mM)	Kcat* (s^−1^)	Kcat/km (M^−1^s^−1^)	Amino acid number	Theoretical isoelectric point	References
*Trypanosoma cruzi*	4880	0.44 ± 0.05	6000 ± 230	1.36 × 10^7^	251	8.64	^18^; This work
*Trypanosoma brucei*	4500	0.45 ± 0.05	5167 ± 117	1.15 × 10^7^	250	9.08	^18^; This work
*Entamoeba histolytica*	2721	0.14 ± 0.03	3649 ± 254	2.5 × 10^7^	261	5.89	^19^; This work
*Homo sapiens*	3908	0.22 ± 0.03	3843 ± 117	1.7 × 10^7^	249	6.45	^20^; This work
*Leishmania mexicana*	4125	0.47 ± 0.01	2800 ± 164	5.9 × 10^6^	251	8.28	^19^; This work
*Plasmodium falciparum*	568	0.81 ± 0.04	5846 ± 39	7.3 × 10^6^	248	6.01	^21^; This work
*Taenia solium*	3920	0.52 ± 0.03	2457 ± 101	4.7 × 10^6^	250	6.60	^22^; This work
*Saccharomyces cerevisiae*	3429	0.79 ± 0.1	8690 ± 142	1.1 × 10^7^	248	6.01	^23^; This work
*Giardia lamblia*	4084	0.1 ± 0.01	3282 ± 103	2.9 × 10^7^	257	7.01	^24^; This work
*Thermus thermophilus*	964	0.06 ± 0.01	1788 ± 0.9	3.0 × 10^7^	250	5.84	This work
*Bovine serum albumin*	—	—	—	—	583	4.90	—
*Horse spleen ferritin type I*	—	—	—	—	174^#^	5.37^#^	—

*The result shown is the average of three independent experiments. ^#^Light chain Genbank: P02791.

### TIMs from several species are able to form native active oligomers

**Figure 2:**
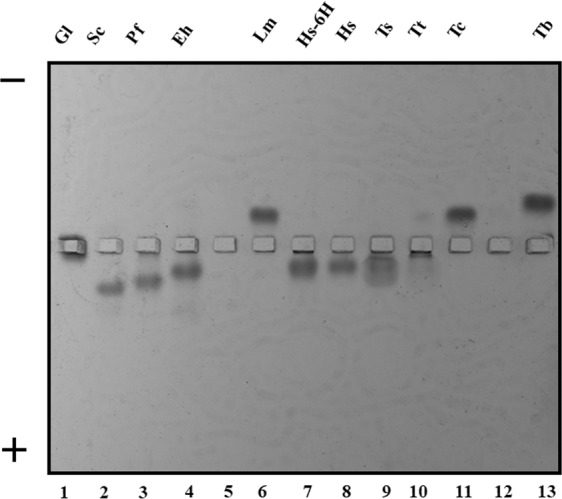
Horizontal clear native electrophoresis showing the polarity-related migration of the TIMs from all species used in this work. The purified enzymes were loaded in the middle of a horizontal 6% acrylamide gel. The name codes are the same used in Fig. 1.

Previously, Swamy *et al*.^17^ detected high molecular weight aggregates (1320 kDa) from rabbit TIM using blue native electrophoresis (BNE). To know if this aggregation process is common for TIM, we analysed samples purified from different species in this native system. We found that TIMs from several species form high molecular weight aggregates that vary in number and molecular masses. Nevertheless, the resolution of the aggregation pattern in this type of gels was not optimal (Fig. S1, Suppl. Information). The TIMs from four species (*Giardia lamblia* TIM: GlTIM, *Trypanosoma brucei* TIM: TbTIM, *Trypanosoma cruzi* TIM: TcTIM and *Leishmania mexicana* TIM: LmTIM) did not enter the gel, even though the presence of Coomassie blue in this system, contributes with negative charges that favour protein migration^25^. The theoretical isoelectric point (Ip) indicates that TbTIM, TcTIM and LmTIM possess the most basic values, while GlTIM has an Ip near to the pH of the buffer used in the system (pH = 7.0) (Table 1). This suggests that the migration of the TIMs in BNE is highly dependent on the charge/mass ratio of the sample. To further investigate this effect, a 7 × 7 cm 6% acrylamide horizontal gel (0.5 cm width) was prepared for the analysis of the samples using native electrophoresis. Figure 2 shows that the migration of the *Saccharomyces cerevisiae* TIM: ScTIM, PfTIM, *Entamoeba histolytica* TIM: EhTIM, *Homo sapiens* TIM: HsTIM, *Taenia solium* TIM: TsTIM and TtTIM, occurs in the cathode-anode direction (normal polarity), but TbTIM, TcTIM and LmTIM migrate in the anode-cathode direction (inverse polarity). In contrast, GlTIM is not able to migrate at all, probably because of its small superficial charge at pH 7.0. Because the protein-dye interaction of the Coomassie blue used in BNE may shift the apparent molecular mass of the aggregates^26^, we decided to analyse the samples using clear native electrophoresis (CN-PAGE), using different polarities.

**Figure 3:**
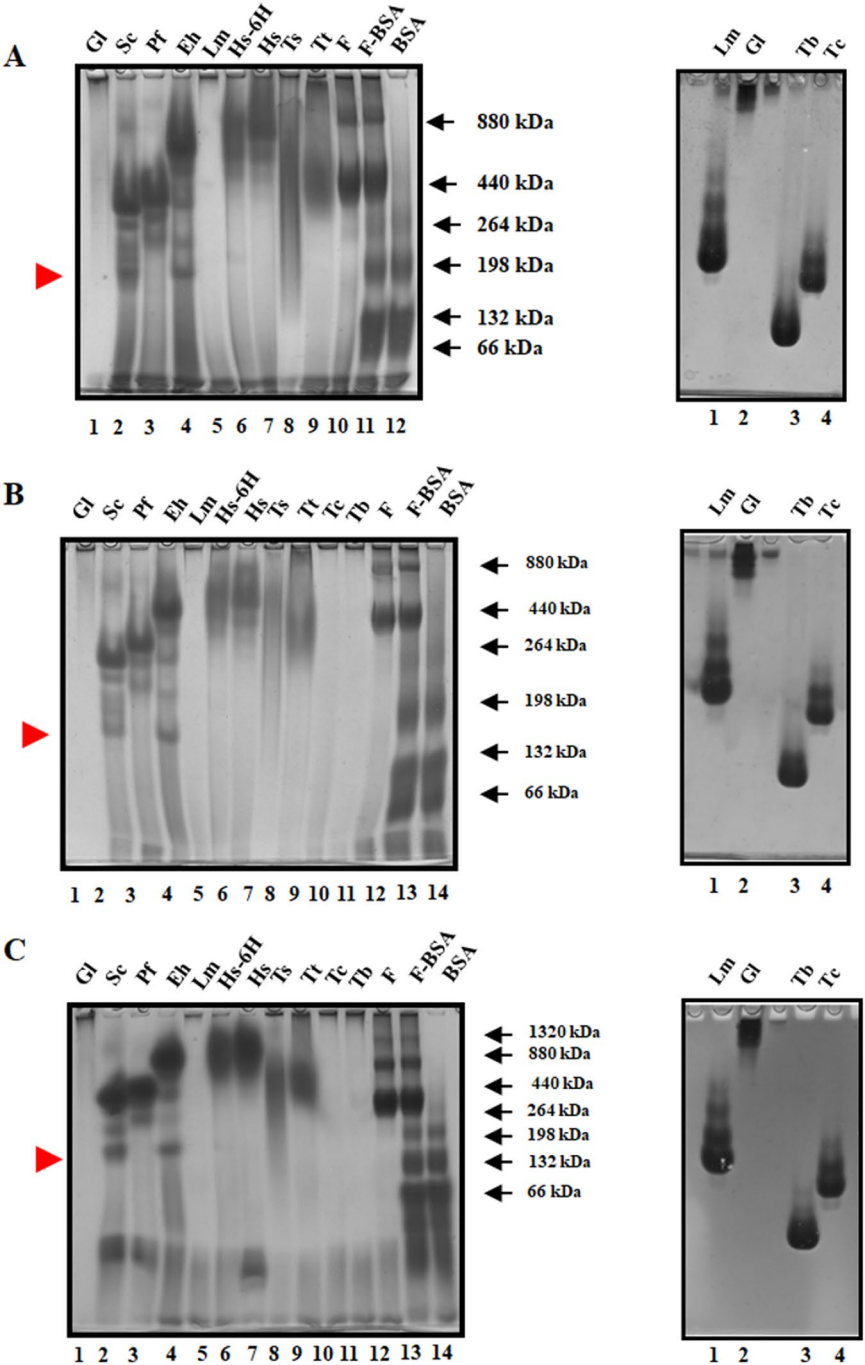
CN-PAGE showing native aggregates of TIM from all species used in this work. TIM aggregates were separated by CN-PAGE according the polarity-related migration determined in Fig. 2, *left panels*: cathode-anode direction (normal polarity) ScTIM, PfTIM, EhTIM, HsTIM, TsTIM and TtTIM, *right panels*: anode-cathode direction (inverse polarity) GlTIM, TbTIM, TcTIM and LmTIM. Different HEPES/imidazole concentrations in the gel buffer are shown. (**A**) 40.0 mM HEPES, 15.3 mM imidazole, (**B**) 88.9 mM HEPES, 34.0 mM imidazole and (**C**) 133.2 mM HEPES, 50.0 mM imidazole. The red arrowheads indicate the small aggregates (3 dimers) of EhTIM and ScTIM. The sizes of the well characterized oligomers of type I ferritin from horse spleen and bovine serum albumin are shown for the gels run in normal polarity. The name codes are the same as those used in Fig. 1.

A modification of the HEPES-imidazole system, used by McLellan^27^, allowed the resolution of aggregates from ScTIM, PfTIM, EhTIM, HsTIM, TsTIM and TtTIM, along with the molecular mass markers used in normal polarity: Type I ferritin from horse spleen (SIGMA) and bovine serum albumin (SIGMA, heat shock fraction) (Fig. 3A left panel). The aggregates of GlTIM, TbTIM, TcTIM and LmTIM were resolved with inverse polarity (Fig. 3A right panel). Both systems, with normal and inverted polarity, were buffered at pH 7.0. To improve the definition of the bands, we varied the concentration of HEPES and imidazole in the gels, keeping the same proportion of both salts. The resolution of the low molecular mass bands increased with increasing concentration of the salts in the gel (Fig. 3B,C). In all cases, the anode and cathode buffers used in the electrophoresis always remained the same (40 mM HEPES and 15.3 mM imidazole).

All the TIMs tested produced oligomers larger than a dimer, although estimating the size of the aggregates was difficult. We identified 2 to 7 TIM aggregates with high molecular masses in the normal polarity system. The two molecular mass markers used, albumin and ferritin, form oligomers of known size, with masses ranging from 66 to 1320 kDa^28^. The molecular masses and the relative migration of these markers and their aggregates was plotted for each gel and all yielded a linear correlation, even at different salt concentrations (mean R^2^ = 0.986) (Figs S2–S4, Suppl. Information). These linear regressions were based on the relative migration of the markers and the molecular masses previously reported for their aggregates. The molecular masses of the aggregates of the TIMs in each gel condition were estimated from these linear regressions. Table 2 shows the mean molecular masses and stoichiometries calculated for the TIM aggregates (Suppl. Tables 1–3, Suppl. Information). Some of the recombinant TIMs were expressed with a tag of 6 histidines (pET-3aHIS and pRSET vectors). To evaluate if this his-tag affects the oligomeric associations, we compared the electrophoretic profiles of HsTIM and HsTIM-6H, and no difference in their association properties was observed (Fig. 3 and Table 2). The aggregates of ScTIM were the largest, with a molecular mass of 1166 kDa, consisting of approximately 21 dimers, followed by aggregates of HsTIM and EhTIM with molecular masses of ~900 kDa (Suppl. Table 3, Suppl. Information). EhTIM and ScTIM also formed small aggregates, consisting of approximately 3 dimers (Fig. 3, *left panels* red arrow heads). A similar distribution in the profile of the aggregates was observed with inverted polarity electrophoresis, where LmTIM, TcTIM and TbTIM form 4, 3 and 3 bands of aggregates, respectively (Fig. 3 right panels). We were unable to calculate the apparent molecular mass of these aggregates, since the markers (ferritin and albumin) do not penetrate the gel when subjected to an inverted polarity. In the case of GlTIM, we could not discern if the absence of migration is due to the small mobility of the high molecular mass aggregates or to the low overall surface charge of the protein at pH 7.0.

**Table 2:** Mean molecular masses and stoichiometries of the TIM aggregates determined by CN-PAGE.

Organism or protein	Number of aggregates	Estimated molecular mass of the aggregates	Number of dimers in the aggregate
*Trypanosoma cruzi*	3	—	—
*Tryapanosoma brucei*	3	—	—
*Entamoeba histolytica*	6	873 ± 83 582 ± 78 472 ± 49 351 ± 16 233 ± 5 163 ± 14	16 10.5 8.5 6.5 4 3
*Homo sapiens*	4	881 ± 142 594 ± 1.4 473 ± 7 405 ± 0	16 11 8.5 7.5
*Homo sapiens 6 H*	4	929 ± 92 638 ± 37 513 ± 20 405 ± 0	17 11.5 9 7.5
*Leishmania mexicana*	4	—	—
*Plasmodium falciparum*	4	795 ± 238 385 ± 27 316.5 ± 5 251 ± 3	14.5 7 6 4.5
*Taenia solium*	2	624 ± 38 461 ± 23	11 8.5
*Saccharomyces cerevisiae*	7	1166 ± 0 677 ± 134 334 ± 25 278 ± 12 225 ± 9 197 ± 4 165.5 ± 8	21 12 6 5 4 3.5 3
*Giardia lamblia*	ND	—	—
*Thermus thermophilus*	1	485 ± 89	9
*Horse spleen ferritin type I* ^*^	3	*1320* *880* *440*	—
*Bovine serum albumin* ^*^	4	264 198 132 66	—

ND. Not determined.

**Figure 4:**
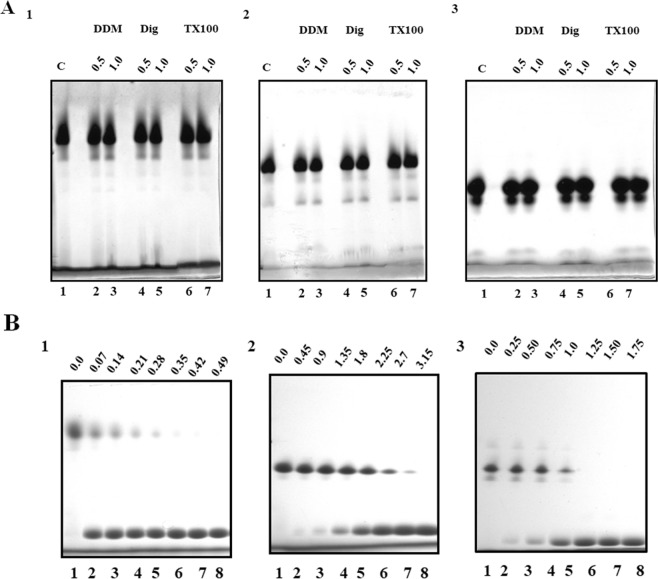
BN-PAGE for the characterization of the stability of some TIM aggregates in increasing concentrations of non-ionic and ionic detergents. (**A**) Thirty micrograms of purified HsTIM (1), EhTIM (2) and PfTIM (3) were loaded with increasing concentrations of n-dodecyl-β-D-maltoside (DDM), digitonin (Dig) and Triton X-100 (TX100). (**B**) Thirty micrograms of purified HsTIM (1), ScTIM (2) and PfTIM (3) were loaded with increasing concentrations of Lithium Dodecyl Sulphate. Detergent concentrations (% m/v) are indicated above each corresponding lane.

To discard possible non-specific, hydrophobic-driven associations due to the exposure of non-polar residues that are normally-hidden, we tested the stability of TIM aggregates from three species using three non-ionic detergents: n-dodecyl-β-D-maltoside, digitonin and Triton X-100. These detergents are used to extract large membrane-embedded complexes, without destroying their native associations^29–32^. Figure 4A shows the stability of the aggregates of HsTIM, EhTIM and PfTIM in the three detergents, which were used in two concentrations (0.5% and 1.0%). No evident dissociation can be observed in any condition, which means that these aggregates are not formed because of non-specific hydrophobic associations, but are due to native interactions between the purified monomers. The thermal stability of aggregates of TbTIM was analyzed following the change in fluorescence of the bound dye SYPRO-Orange. No evident change in the fluorescence signal could be observed in the initial points (up to 45 °C), indicating that the freshly purified enzyme does not expose hydrophobic surfaces (Fig. S5, Suppl. Information). We also tried to dissociate HsTIM, EhTIM and PfTIM aggregates with increasing concentrations of Lithium Dodecyl Sulphate (LDS). This SDS-analogue is suitable for electrophoresis at low temperature and low pH^33,34^. All the aggregates from the analysed TIMs can be dissociated with low LDS concentration, even though they differ in the amount necessary to complete their dissociation into single monomers (Fig. 4B). Thus, TIM aggregates have different structural stabilities, which form reversibly and do not involve covalent bonds (*e.g*. disulphide bonds). To confirm the latter, purified ScTIM, PfTIM, EhTIM and HsTIM were incubated 30 min at 25 °C in presence of 10 mM of Dithiothreitol (DTT) and subjected to CNE. No evident difference can be observed in the electrophoretic profiles of the aggregates, indicating that no apparent disulphide bonds are formed in the native aggregation process of these TIMs (Fig. S6, Supp. Information). Only GlTIM has been reported to form disulphide bonds between a cysteine partly exposed to the medium (C202)^35^. However, to avoid this problem we used GlTIM C202A, a mutant unable to form disulphide bonds^35^.

**Figure 5:**
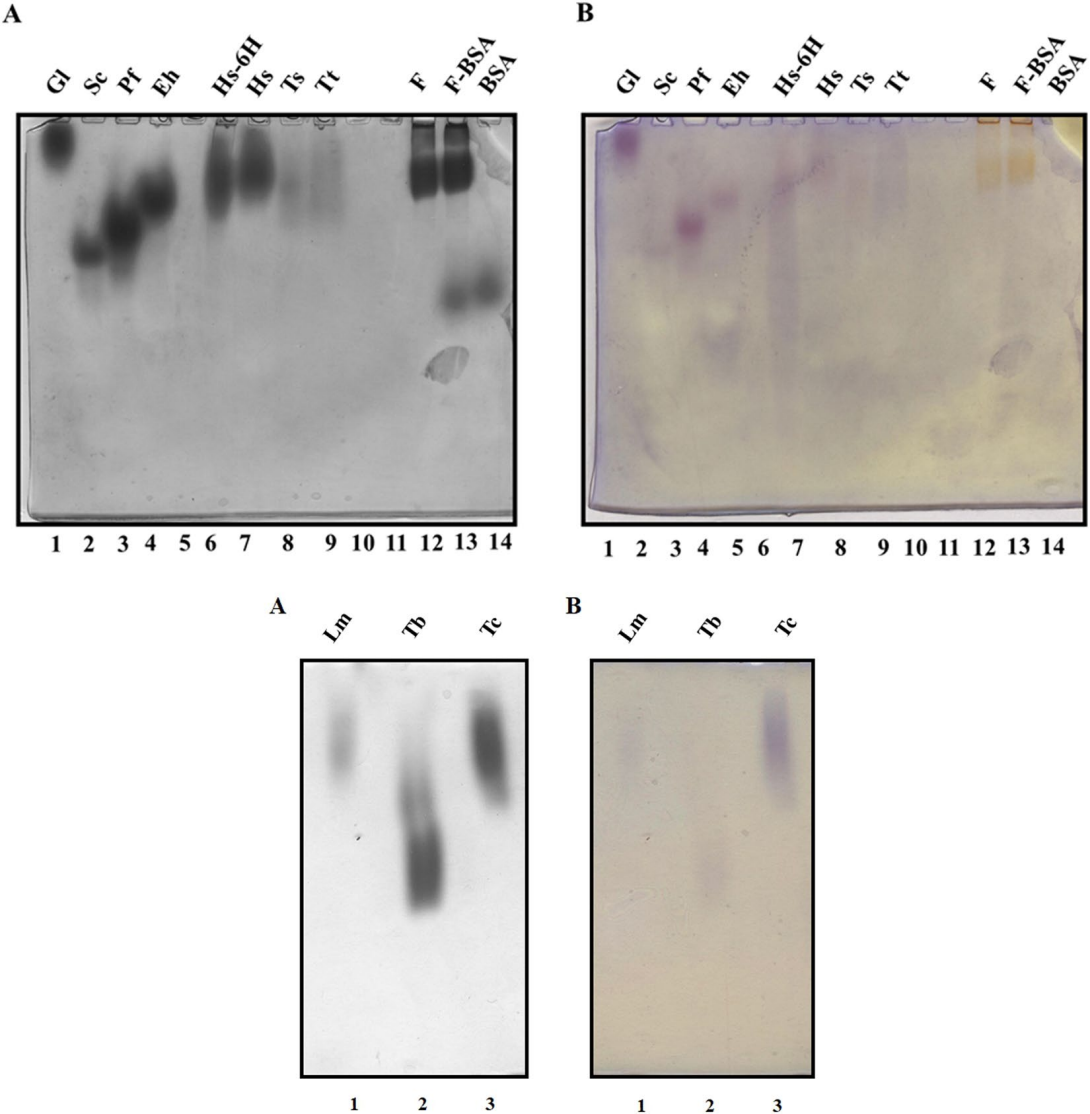
*In-gel* activity of the native TIM aggregates from all species used in this work. *Left panels*: Coomassie blue stained gels of the TIM aggregates separated in normal polarity (*upper panel*, **A**) and inverse polarity (*lower panel*, **A**). *Right panels*: TIM activity stain of the native aggregates for the corresponding gels in the left panels (*upper and lower panels*, **B**). The name codes are the same as those used in Fig. 1.

In the case of TIM, the catalytic activity is one of the parameters that can indicate how well or how poorly the enzyme is folded^36^. To elucidate if TIM, forming high molecular complexes, is able to perform catalysis, we determined *in-gel* activity, after separating the aggregates by CN-PAGE. Since TIM is inactive in the HEPES-imidazole buffer, the gels with the separated enzymes were incubated for 1.5 h in a developing buffer (see material and methods section 3.5). All aggregates were active and transformed MTT into formazan, even though there are differences in the intensities of the bands for different species (Fig. 5). This indicates that TIM oligomerization is not due to partial unfolding of the protein, and that these high molecular aggregates are catalytically active.

### Stable TIM aggregates can be separated by liquid chromatography and their size can be estimated with DLS

**Figure 6:**
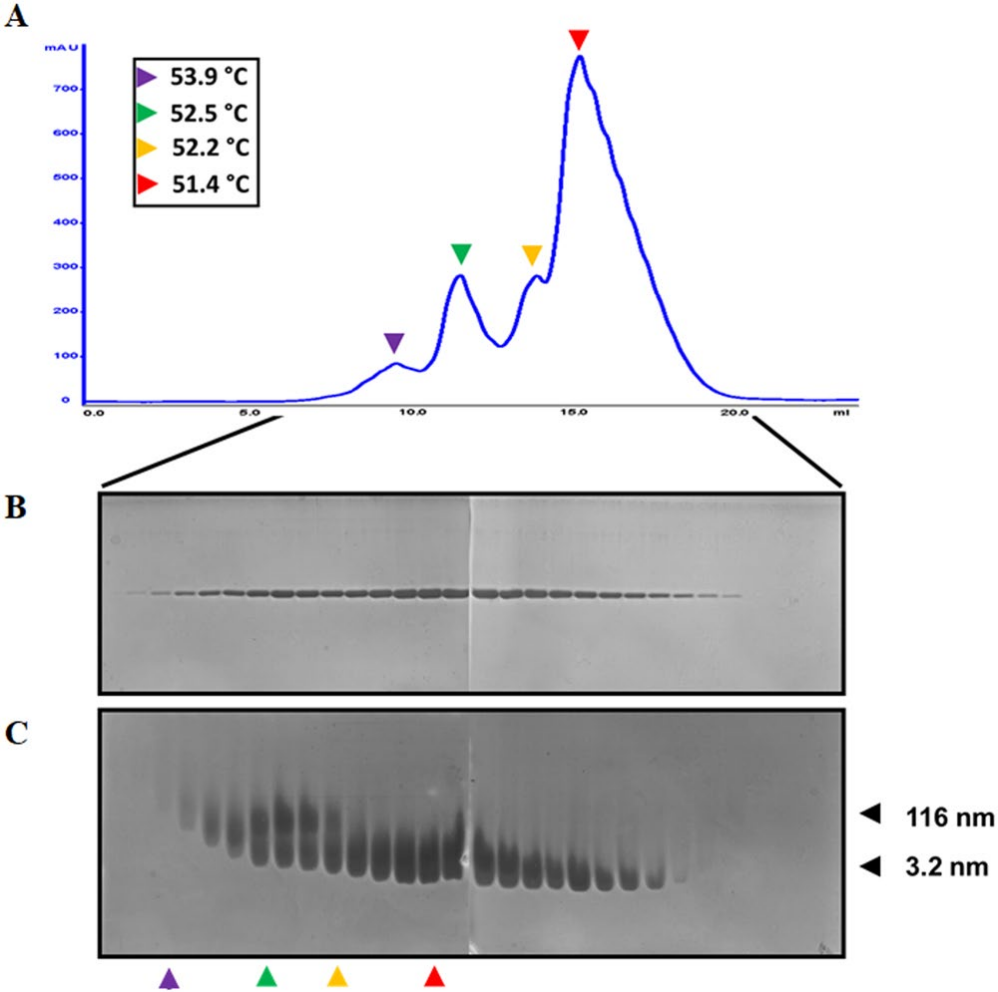
Separation of the aggregates of TbTIM by ion exchange chromatography. A total of 4.7 mg of TbTIM were loaded onto a Source 15 column. (**A**) UV signal from the elution pattern of the chromatography (blue line) applying a 50–100 mM NaCl gradient, four peaks are observed (colour arrow heads). *Insert*: Melting temperature of the four fractions. (**B**) SDS-PAGE gel of the fractions from the elution. (**B**) CN-PAGE of the fractions from the elution. The hydrodynamic radii of the different TIM species are shown to the right.

Stable high molecular mass protein oligomers can be separated by different techniques^37^; however, in some cases, meta-stable oligomers can be formed, whose half-life can last from a few seconds to hours. Depending on the association and dissociation constants of the oligomers, these may be detected with some particular analytical method^9^. In gradient gels, proteins migrate through several networks of pores of different sizes; the movements the enzymes undergo and the obstacles they encounter during their migration, could favour their aggregation. Another factor could be the duration of the electrophoresis. To discard that aggregates of TIM are formed as artifices of gel electrophoresis and to characterize them further, a sample of TbTIM (from the SP Sepharose fast flow exchange column) was separated by an analytical Source 15 S column (with a matrix volume of 1 mL). This new separation yielded four different peaks, which eluted at different concentrations (between 50 and 100 mM) of NaCl (Fig. 6A, *colour arrow heads*). SDS-PAGE analysis of the eluted fractions revealed that all are pure TbTIM (Fig. 6B). The analysis of the elution fractions by CN-PAGE showed the mix of mainly two different species in different proportions (Fig. 6C). The specific activity of the four peaks was 5620, 5300, 5160 and 4999 µmol mg^−1^ min^−1^, respectively. These values agree with the specific activity shown in Table 1, confirming that the mixes of TIM aggregates are active, with nearly equivalent enzymatic activities.

The reversible and rapid equilibrium of self-association, can be evidenced in separations of a purified protein by size exclusion chromatography or sedimentation, resulting in the appearance of multiple peaks formed by mixtures of oligomers^9^. Nevertheless, in our hands the TIM oligomers could not be isolated using gel filtration chromatography. We could only isolate dimers and monomers, even though this technique has shown an excellent resolution between monomers that differ by only one residue^38^. We also performed an analysis of particle size using dynamic light scattering (DLS) spectroscopy for the peaks from the Source column. Previous work has shown that the hydrodynamic radius determined by size exclusion chromatography and pulse field gradient NMR for ScTIM is 2.4 and 2.9 nm, respectively^39–41^. Our DLS analysis of TbTIM showed mainly two types of particles with hydrodynamic radii of 3.2 ± 0.3 and 116.3 ± 12 nm, which may correspond to aggregates of 1 and 38 dimers respectively (Fig. 6C). Additionally, the temperature of denaturation (Tm) of each peak was determined using thermal shift assay. The largest species had a Tm 2.5 °C higher than the smallest species (Fig. 6A, *insert*), indicating a correlation between structural stability and size of the aggregates.

### Time favours the formation of non-reversible fibrillar aggregates in TbTIM

**Figure 7:**
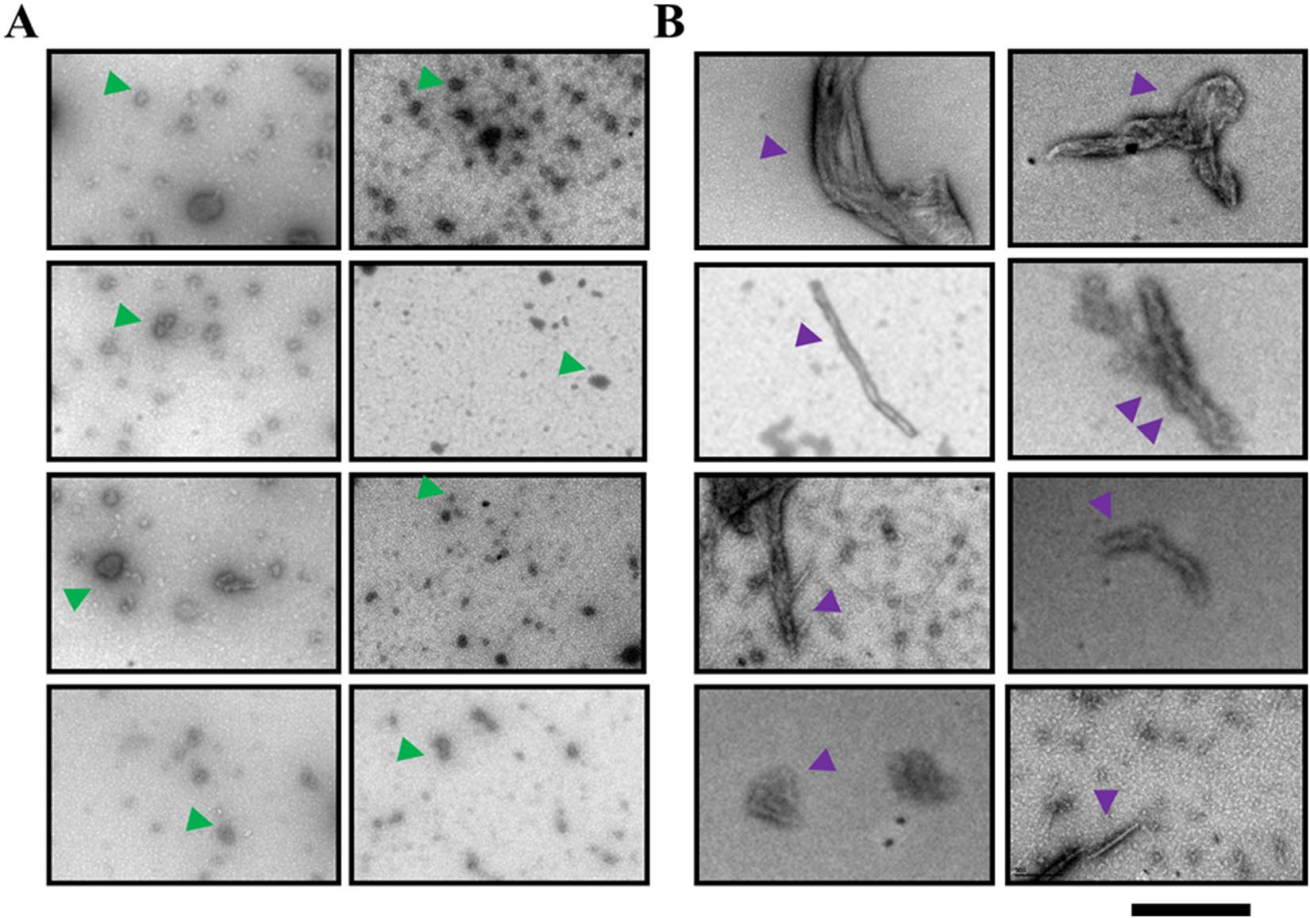
Electron microscopy images of the aggregates of TbTIM. (**A**) Images of samples from freshly purified TbTIM (*left panels*). Oligomers of different sizes are indicated (green arrow heads). (**B**) Images of samples of TbTIM 20 days after purification (*right panels*) showing greater aggregates and fibrillar structures (purple arrow heads). The black line represents 200 nm.

In order to visualize the nature of TIM aggregates in more detail, we analysed two kinds of purified TbTIM by transmission electron microscopy (TEM): one corresponding to freshly purified protein, and the second with protein stored at 4 °C for 20 days. The microscopic images revealed the presence of multiple globular aggregates with sizes ranging from 20 to 100 nm (corresponding to aggregates of 6 to 30 dimers) (Fig. 7A, green arrow heads). Higher oligomers and fibrillar aggregates appeared with time in the stored sample (Fig. 7B, purple arrow heads), and are characteristic of amyloid fibril formation processes^42,43^. The accumulation and deposition of non-covalent homopolymers of proteins is associated with more than 25 pathologies, which include Alzheimer’s disease, Parkinson’s disease, Huntington’s disease, and type II diabetes (reviewed in^16^).

**Figure 8:**
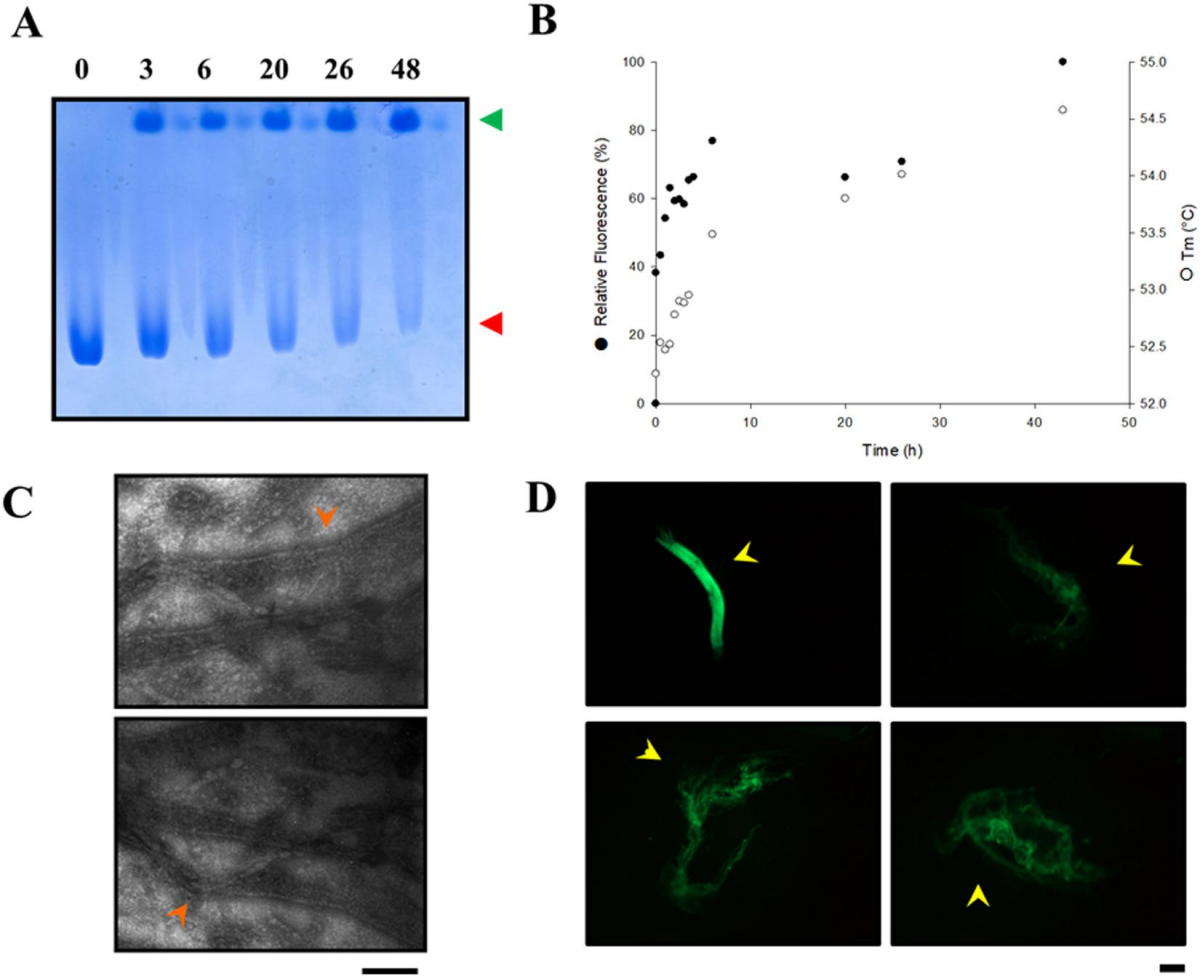
Induced amyloid fibrillar TbTIM aggregates by heme peroxidase–H_2_O_2_–NO^2−^. Freshly purified dimeric enzyme was incubated with heme peroxidase–H_2_O_2_–NO^2−^ (**A**) CN-PAGE from different incubation times (in hours) with the oxidative/nitrative stress agent. *Red arrow head*: dimeric TbTIM, *green arrow head*: horseradish peroxidase band. The numbers signalled the incubation time. (**B**) Denaturing temperature (Tm) and SPYRO orange fluorescence at different incubation times. (**C**) TEM images of TbTIM incubated 24 h with the oxidative/nitrative stress agent. Large characteristic fibrillar structures (orange arrow heads) are observed. The black line represents 200 nm. (**D**) Fluorescence microscopy images from fibrillar amyloid TbTIM aggregates stained with Thioflavine T prepared by 48 h incubation with the oxidative/nitrative stress agent. The black line represents 100 nm.

TIM is one of the highly nitrotyrosinated proteins in Alzheimer’s disease^14,44^. To further characterize the capacity of TbTIM to form fibrillar aggregates, freshly purified dimeric enzyme was incubated at 37 °C in presence of an oxidative/nitrative stress agent (heme peroxidase–H_2_O_2_–NO^2−^)^13^. At different times samples were taken and analysed by CN-PAGE. Figure 8A, (*red arrow head)* shows the decrease of free dimers, at increasing times of exposure to the stress agent, indicating the formation of high molecular aggregates, which probably do not enter the gel. The additional band is observed on top of the lane, whose intensity remains apparently constant in time, may correspond to the horseradish peroxidase, present in this assay, which has little or no migration in the gel of inverse polarity (Fig. 8A, *green arrow head*). To follow the formation of the high molecular weight aggregates of TIM, the fluorescence of the dye SYPRO orange and the variation of the Tm value were measured for aggregates (Fig. 8B). This dye binds to amylin amyloid fibrils, but not to pre-fibrillar intermediates^45^, therefore, the increase of the fluorescence indicates the fibrillary aggregation of TbTIM. In addition, because the amyloid fibrillar aggregates have high temperature stability^46^ an increase in the Tm value is correlated to the native aggregation process, as shown in Fig. 6. Furthermore, typical fibrillar aggregates were observed by negative staining using electron microscopy after 24 h incubation (Fig. 8C, *orange arrow heads*). Finally, larger fibrillar aggregates (>1 µm), following a 48 h incubation, were stained with thioflavin T and observed under UV light in a fluorescence microscope (Fig. 8D). Thioflavine T interacts with a variety of amyloid fibrils in suspension, generating an amyloid-specific fluorescent signal^47–49^. Taken together, these data clearly show the propensity of TbTIM to form fibrillar amyloid aggregates. This conversion of the free dimeric form into fibrillar aggregates is directly related to the loss of enzymatic activity. This process is clearly accelerated in presence of oxidative/nitrative stressing conditions, giving rise to large fibrillar aggregates that are evident in TEM images after 24 h incubation (Fig. 8C), and that exhibit larger sizes than the fibrillar aggregates formed by the cold-stored enzyme (>360 h) (Fig. 7B). Additionally, the size of the stress-induced fibrillar aggregates is considerably larger (>1 µm) as judged by fluorescence microscopy (Fig. 8D). In general, it has been proposed that all peptides have the capability to form amyloid fibers, nevertheless, the propensity to form these kind of structures depends on multiple factors like charge, sequence, hydrophobicity, etc.^50^. Most of the amyloid fibers precursor peptides have highly unstable structures and are considered intrinsically disordered proteins^16^. Nevertheless, TIM possesses a defined structure and a high capacity of self-association that forms two kind of aggregates: native globular reversible aggregates and amyloid fibrillar aggregates *in vitro*. In contrast, nitrotyrosinated TIM associates with Tau and Abeta peptides to start the fibrillar association *in vivo*^14,44^.

### Bioinformatic search of regions prone to aggregation in TIM

Based on experimental information and computer-based approaches, multiple aggregation-prone amylogenic regions are present in many proteins^51^ and have been identified in several sequences of TIM. Here, the amylogenic regions in the TIMs studied were predicted using the WALTZ^52^ algorithm, a program that calculates the propensity to form parallel or antiparallel beta fibrillar structures for a given sequence. To analyse the amylogenic zones, the sequences of all TIMs were divided into eight regions, as previously reported by Rodríguez-Bolaños *et al*.^18^. Three conserved amylogenic regions were predicted for regions 2, 3 and 6 (Fig. S7 Suppl. Information); the first region extends from residue 33 to 51, and is formed by external loop 1, beta sheet 2, internal loop 2 and the beginning of alpha helix 2; the second region includes residues 63 to 73, corresponding to external loop 2; and the third region extends from residues 59 to 69, and includes the beta sheet 6, internal loop 6 and the start of alpha helix 6 (Fig. S8 Suppl. Information). It is also known that regions 1, 2 and 3 of TbTIM and TcTIM are involved in dimerization. The internal loops play a fundamental role in the correct positioning of the monomers, and are therefore also related to self-association^53^. It has also been observed that external loops 1 and 2 have an important role in the aggregation of the enzyme. Interference in communication between external loop 1 and the amino-terminus of helix 2, and of external loop 2, has been reported to promote aggregation of TcTIM and TbTIM after refolding^18^. In addition, previous studies described the presence of three regions in some TIM sequences that have some similarity (approximately 8–22%) with Abeta. Of these segments, the one with the highest similarity corresponds to amino acids 173 to 213 (in the sequences of *Escherichia coli* and *Culex tarsalis*) which have 20% identity with Abeta. This isolated fragment is capable of forming amyloid aggregates^54^. There are a few reports about proteins with the TIM barrel motif that form fibrillary aggregates, however, it has also been described that HsTIM forms amyloid deposits in patients with Alzheimer’s disease^14,51,55^. The sequences of TbTIM and HsTIM share 50% identity and also exhibit 15 and 18% identity with Abeta, respectively (Suppl. Tables 5 and 6, Suppl. Information). Overall, the alignment between Abeta and the sequences of the TIMs studied shows an identity that ranges between 15 and 25% (Suppl. Table 5, Suppl. Information). GlTIM, LmTIM, EhTIM and TcTIM have the highest identity scores, 25, 23, 20 and 20%, respectively. The area where this similarity is found corresponds mainly to the hinged “lid” loop or catalytic loop, and in all cases consists of regions 6 and 7 which correspond to helix 6, external loop 6, beta sheet 7 and internal loop 1. This could possibly indicate that the fibrillary aggregation process is conserved among the TIMs from different species.

#### Concluding remarks

The formation of reversible aggregates by the self-association capacity of TIM, could favour their adaptation to some particular changes in the cell or in the extracellular environment. All TIMs we studied are capable of forming oligomers of different sizes, and their aggregation profiles appear to be influenced by their surface charges, that give rise to different groups of heterogeneous aggregates. It has recently been described that TIM has multiple functions in addition to its participation in glycolysis (reviewed in^56^); this self-associative capacity could regulate some of its non-catalytic activities. In other pathological situations, the presentation of protein aggregates to the immune system could trigger the formation of autoantibodies^57^. TIM has been described as a potent allergen, which may also be due to its ability to self-associate. Since TbTIM is able to form fibrillary irreversible aggregates like HsTIM, this may be a conserved feature among triosephosphate isomerases.

## Material and Methods

### Heterologous expression of the TIMs from several species

The TIM genes from *Entamoeba histolytica* (EhTIM), *Giardia lamblia intestinalis* mutant C202A (GlTIM), *Leishmania mexicana* (LmTIM), *Plasmodium falciparum* (PfTIM), *Taenia solium* (TsTIM), *Homo sapiens* (HsTIM), *Saccharomyces cerevisiae* (ScTIM), *Trypanosoma cruzi* (TcTIM), *T. brucei* (TbTIM) and *Thermus thermophilus* (TtTIM) were cloned into the plasmids pRET (EhTIM), pET-3a (TcTIM, TbTIM), pET-3aHIS (HsTIM, GlTIM, TtTIM, LmTIM), pKK233-3 (ScTIM), pRSET (TsTIM) and pTRC99A (PfTIM). Each plasmid with the corresponding TIM gene was transformed into *E. coli* Bl21 (DE3)-p-Lys S cells.

Each transformant was grown at 37 °C in Luria-Bertani medium supplemented with 100 µg/mL ampicillin until reaching an optical density of 0.6 at 600 nm. After this time, the cultures were induced with 0.4 mM (TbTIM and TcTIM) or 1 mM (EhTIM, GlTIM, LmTIM, PfTIM, TsTIM, HsTIM, ScTIM and TtTIM) of isopropyl β-D-thiogalactopyranoside (final concentration) and incubated for an additional 12 hours at 30 °C to induce the corresponding proteins before harvesting the cells (6400 × *g*/15 min).

### Purification of EhTIM, GlTIM, LmTIM, PfTIM, TsTIM, HsTIM, ScTIM and TtTIM

After harvesting, cells were suspended in 30 mL of lysis buffer (20.0 mM Tris, 1.0 mM EDTA, 2.0 mM DTT, pH 8). Each suspension was sonicated for 1 min at a power setting of 5 W, with a 2 min rest, for a total of 10 cycles, and then centrifuged for 15 min at 200 000 × *g* and the supernatant recovered. Later, solid ammonium sulphate ((NH_4_)_2_SO_4_) was carefully added to a 90% w/v final concentration, and the sample was incubated at 4 °C with gentle agitation for 12 h. The sample was centrifuged for 15 min at 35 000 × *g* and the resulting pellet was suspended in 3 mL of 100.0 mM triethanolamine (TEA), 10.0 mM EDTA, pH 7.4 and sufficient (NH_4_)_2_SO_4_ was added to reach a final concentration of 2.2 M. Immediately afterwards the protein was loaded onto the TOYOPEARL® Butyl -650M  hydrophobic interaction column (GE Healthcare, 30 mL column volume), previously equilibrated with the same buffer, but containing 2.0 M (NH_4_)_2_SO_4_. Protein elution was performed using a 2.0 to 0 M gradient of (NH_4_)_2_SO_4_. Four millilitre fractions were collected and analysed by SDS-PAGE; those containing TIM were pooled and dialyzed overnight against 3 L 100.0 mM TEA, 10.0 mM EDTA, pH 7.4 at 4 °C. After removal of the precipitated material (35 000 × *g*/30 min) the samples were loaded onto a Source 15Q anion exchange column (24 mL column volume), previously equilibrated with the same buffer. Protein elution was performed with a linear gradient of 0–500 mM NaCl. Four mL fractions were collected and analysed by SDS-PAGE; those fractions enriched with TIM were pooled and concentrated with an Amicon Ultra-15 Centrifugal Filter 10 kDa (EMD Millipore) to a final volume of 5 mL, and then precipitated with 70% (NH_4_)_2_SO_4_ and kept at 4 °C.

### Purification of TcTIM and TbTIM

The cell pellet was suspended in 30 mL of lysis buffer (50.0 mM MES, 300.0 mM NaCl, 1.0 mM DTT, 0.5 mM EDTA, pH 6.3). The sample was sonicated for 1 min at a power setting of 5 W, with a rest of 2 min, for a total of 10 cycles. This suspension was centrifuged for 60 min at 200 000 × *g*. The supernatant was diluted with buffer A (50.0 mM MES pH 6.3) to a final concentration of 20.0 mM NaCl. The sample was then loaded onto a SP Sepharose fast flow column (30 mL volume column, GE Healthcare), previously equilibrated with buffer A. Protein elution was performed using a linear gradient of 0–500 mM NaCl. Three mL fractions were collected and analysed by SDS-PAGE. Those fractions enriched with TIM were pooled and (NH_4_)_2_SO_4_ was gradually added to reach the final concentration of 70% (w/v). The sample was incubated with gentle agitation for 16 hours at 4 °C. After this step, the purification with the Butyl toyopearl column and the subsequent (NH_4_)_2_SO_4_ precipitation were performed as described in section 3.2.

All the precipitated purified enzymes were dialyzed against 2 L of 100 mM TEA, 10 mM EDTA, pH 7.4, just before use. Protein concentration measured reading the absorbance at 280 nm, and using a molar extinction coefficient ε = 34950 M^−1^ cm^−1^ ^58^.

### Native electrophoresis

Horizontal clear native electrophoresis was performed in a 7 × 10 Gel Box chamber (Labnet Int.). A 7 × 7 cm 6% acrylamide horizontal gel (0.5 cm width) with sample wells located in the middle of the gel was prepared, to allow migration of the samples in both directions during the electrophoretic separation. The buffers for the gels and the electrophoresis were 40.0 mM HEPES and 15.3 mM imidazole. The electrophoresis was performed at 80 V during 1 h at 4 °C. Clear native polyacrylamide gel electrophoresis (CN-PAGE) was performed using a Mini-PROTEAN II protein electrophoresis chamber (BIO-RAD). Only one gel was used for each run. All separations were carried out at a constant voltage of 150 V at 4 °C for 5 hours. The upper and lower reservoirs were filled with fresh buffer containing 40.0 mM HEPES and 15.3 mM imidazole. Before each run, the chamber with the gel and buffers was brought to the desired temperature. The gradient gels used had 4 to 10% polyacrylamide. For some experiments, different HEPES-imidazole ratios were used in the gels, but the concentration of the buffer in the anode and cathode chambers was always the one mentioned above. The HEPES and imidazole concentrations in the gels were as follows: HEPES 40.0 mM, imidazole 15.3 mM, or HEPES 88.9 mM, imidazole 34.0 mM, or HEPES 133.2 mM imidazole 50 mM. Ten micrograms of each purified enzyme were mixed with 5 µL of 16% (v/v) glycerol in 100 mM TEA, 10 mM EDTA, pH 7.4 in a total volume of 30 µL; these samples were the loaded onto each lane.

Blue native electrophoretic separation (BNE-PAGE) under native conditions was performed as reported by^59^ using an acrylamide gradient of 4 – 12%. One microliter of 5% Coomassie Serva blue G solution was added to the sample before loading the gel. Separation was performed at 4 °C and the electrophoretic front was monitored and not allowed to leave the gels.

After migration, all gels were fixed with 50% methanol and 10% acetic acid solution for 15 min and stained with 50% ethanol, 10% acetic acid and 0.1% Coomassie R-250 blue solution for 3–4 h with constant agitation. The gels were destained for 5–6 h with 10% acetic acid solution until the background was clear.

### Determination of *in vitro* and in-gel enzymatic activity

*In vitro* catalytic activity was measured following the inter-conversion of glyceraldehyde 3-phosphate (GAP) to dihydroxyacetone phosphate (DHAP) at 25 °C with the aid of a coupled enzymatic reaction with glyceraldehyde phosphate dehydrogenase (αGDH). The activity was followed by changes in absorbance at 340 nm due to the oxidation of NADH. The reaction mix contained 100.0 mM TEA, 10.0 mM EDTA, 1 mM GAP, 0.2 mM NADH and 20 µg/mL α-glycerol phosphate dehydrogenase. The reaction was measured with 1–5 ng/mL of protein. The determination of the kinetic parameters Km and Vmax was performed by fitting the initial velocity values, determined at different GAP concentrations (0.3 to 3 mM), to a Michaelis-Menten equation.

For the determination of the activity *in-gel*, the enzymatic reaction was measured in the direction of DHAP to GAP. Enzyme activity was detected by changes in the oxidation of NAD^+^ using a coupled colorimetric reaction (dimethyl thiazolyl diphenyl tetrazolium (MTT) assay). After the electrophoresis, the gels were incubated for 1.5 h at 4 °C in developing buffer (50.0 mM Tris, 5.0 mM MgSO_4_ and 5.0 mM NaH_2_AsO_4_•7H_2_O, pH 7.6). After this time the gels were carefully placed in a glass container and uniformly impregnated with a solution containing 11.0 mM phenazine methosulphate (PMS), 19.3 µM MTT, 7.5 µM NAD^+^, 9.8 mM DHAP and 0.2 mg glyceraldehyde 3-phosphate dehydrogenase (GAPDH). The gel was carefully incubated for 2 to 5 min, in darkness, avoiding the formation of bubbles between both surfaces. To fix the colour developed by the formation of formazan, the gel was placed in a container with 7.0% acetic acid for 2 h.

### Separation of TIM aggregates by liquid chromatography

The protein was purified by the SP Sepharose fast flow exchange column, as described in section 3.3. Fractions enriched with TIM were pooled and dialyzed overnight against 3 L of 50.0 mM MES, pH 6.3 at 4 °C. The dialyzed enzyme was loaded onto a Source 15S column (column volume 1 mL) previously equilibrated with the same buffer. Prior to the elution of the protein, the column was washed with 5 volumes of the same buffer. The elution was performed with a linear gradient of 30–125 mM NaCl in the same buffer. The specific activity of the fractions with the highest UV absorbance in each elution peak was measured. Additionally, the purity and the state of aggregation of the fractions were monitored by CN-PAGE and SDS-PAGE.

### Estimation of the molecular weight of TIM aggregates by dynamic light scattering

The dynamic light scattering was performed in a Zetasizer Nano-Zs spectrum (Malvern Instruments, Ltd., U. K.). A 500 µL sample was deposited in the cuvette, and five measurements of each sample were made with 10 scans for each of them. Filtered (PVDF, 0.22 μM) 50.0 mM MES, pH 6.3, was used as a blank.

### Microscopic visualization of TIM aggregates

#### Transmission electron microscopy

Nine microliter aliquots were adsorbed for 5 min onto freshly glow-discharged carbon-coated copper grids. Excess amount of sample was blotted with filter paper and the grids allowed to dry for another 5 min. The grids were stained with 2% uranyl acetate for 5 min followed by 10 min of drying. Images of the TIM aggregates were observed and photographed with Transmission Electron Microscope JEM-1200X EX II (JEOL, Japan) coupled to a CCD GATAN camera.

#### Fluorescence microscopy

TbTIM fibrillar aggregates were stained with 1 mM Thioflavin T in a 1:1 (protein:Thioflavin T) proportion. The sample was incubated 10 min in the dark and observed under blue light in a Nikon SMZ1500 fluorescent microscope. The image acquisition was performed with the NIS-Elements F software (Nikon, Japan).

### Formation of TbTIM fibrillar amyloid aggregates by peroxynitration

The peroxynitrite production was performed as described previously^13^. Briefly, purified TbTIM (0.4 mg/mL) was incubated in 50 mM MES pH 6.3 with 20 µM radish heme peroxidase (Sigma), 1 mM H_2_O_2_ and 1 mM NaNO_2_, at 37 °C with constant agitation (250 rpm). Samples were taken at different times and analysed by CN-PAGE, thermal shift assay and their enzymatic activity was determined.

The thermal shift assay was performed by following the fluorescence of the SYPRO-orange dye in a Real-Time PCR StepOnePlus System (Applied Biosystems, Massachusetts, USA). Each sample (8.4 µg) was loaded onto a 96-well plate and mixed with a 1:1000 dilution of SYPRO-orange dye in 50 mM MES pH 6.3 in a final volume of 20 µL. The melting curve was obtained using a 25–99 °C temperature gradient, with 490 nm as the exciting light wavelength and 575 nm as the detection wavelength.

### Prediction of regions prone to aggregation in the TIM sequences

The prediction of regions prone to aggregation was made using the WALTZ program^52^ (http://switchlab.org/bioinformatics/waltz). These regions were calculated for all TIM sequences using a high-specificity limit to prevent false positives. Sequence alignments were performed with Clustal Omega^60^ (https://www.ebi.ac.uk/Tools/msa/clustalo/).

## Supplementary information

Supporting Information.
