# Supplementary material for: Native aggregation is a common feature among triosephosphate isomerases of different species
Source: Sci Rep. 2020 Jan 28;10:1338. doi: 10.1038/s41598-020-58272-4 (PMC6987189; doi:10.1038/s41598-020-58272-4)
Supplement: Supplementary file 1 — Supporting Information. [file 41598_2020_58272_MOESM1_ESM.docx]

**Native aggregation is a common feature among triosephosphate isomerases of different species.**

Mónica Rodríguez-Bolaños^1^, Héctor Miranda-Astudillo^2^, Edgar Pérez-Castañeda^1^, Diego González-Halphen^2^, Ruy Perez-Montfort^1*^

**Supplementary Information**

**
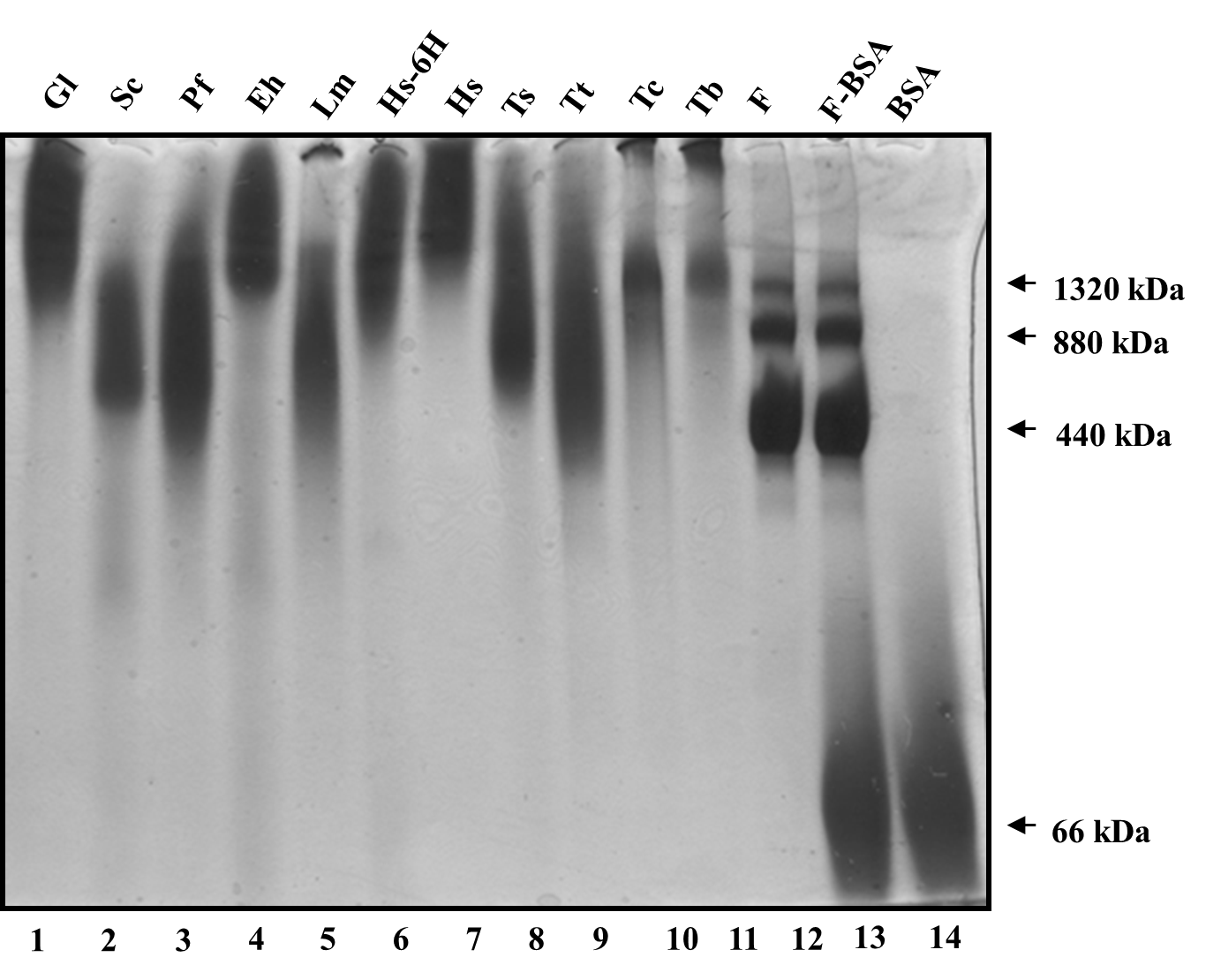
**

**Figure S1. Blue Native PAGE of TIMs from different species showing smears containing aggregates.** 30 µg of each purified enzyme was added with 1 µL of 5% Serva Coomassie Blue solution. The samples were loaded in a 4 - 12% acrylamide gradient gel and separated at 150 Volts for 5 hrs at 4 °C.

**Supplementary Table 1.** Properties of the aggregates of TIM observed using CN-PAGE

(HEPES 40 mM, imidazole 15.3 mM)

| **Organism or protein** | **Number of aggregates** | **Estimated molecular mass of the aggregates** | **Number of dimers in the aggregate** | **Relative distribution**  **(%)** |
| --- | --- | --- | --- | --- |
| *Trypanosoma cruzi* | 3 | - | - | 9  17  74 |
| *Trypanosoma brucei* | 3 | - | - | 6  30  64 |
| *Entamoeba histolytica* | 5 | 672  506  365  238  173 | 12  9  6.5  4.5  3 | 58  2  18  8  16 |
| *Homo sapiens* | 3 | 762  593  468 | 14  10  8.5 | 63  27  10 |
| *Homo sapiens 6H* | 4 | 894  612  499  205 | 16  11  9  4 | 72  9  9  10 |
| *Leishmania mexicana* | 4 | - | - | 7  19  11  63 |
| *Plasmodium falciparum* | 4 | 1056  402  313  249 | 19  7  6  4.5 | 7  79  2  12 |
| *Taenia solium* | 1 | 445 | 8 | 100 |
| *Saccharomyces cerevisiae* | 6 | 805  357  286  234  200  171 | 14.5  6.5  5  4  3.5  3 | 10  70  6  2  1  11 |
| *Giardia lamblia* |  | - | - |  |
| *Thermus thermophilus* | 1 | 428 | 8 | 100 |
| Horse spleen ferritin type I ^*^ | 2 | *880*  *440* | - | 16  84 |
| Bovine serum albumin ^*^ | 4 | 264  198  132  66 | - | 3  25  34  38 |

^*^ Taken from ^1^

**Supplementary Table 2.** Properties of the aggregates of TIM observed using CN-PAGE

(HEPES 88.88 mM, imidazole 34 mM)

| **Organism or protein** | **Number of aggregates** | **Estimated molecular mass of the aggregates** | **Number of dimers in the aggregate** | **Relative distribution**  **(%)** |
| --- | --- | --- | --- | --- |
| *Trypanosoma cruzi* | 3 | - | - | 7  24  69 |
| *Trypanosoma brucei* | 3 | - | - | 12  13  77 |
| *Entamoeba histolytica* | 6 | 814  542  437  334  233  153 | 15  10  8  6  4  3 | 2.5  44  2.5  11  8  32 |
| *Homo sapiens* | 4 | 843  595  478  405 | 15  11  9  7 | 16  62  17  5 |
| *Homo sapiens 6H* | 4 | 860  664  528  430 | 16  12  10  8 | 15  63  18  4 |
| *Leishmania mexicana* | 4 | - | - | 3  17  14  66 |
| *Plasmodium falciparum* | 4 | 739  398  320  253 | 13.5  7  6  4.5 | 5  64  8  23 |
| *Taenia solium* | 2 | 597  451 | 11  8 | 83  17 |
| *Saccharomyces cerevisiae* | 6 | 690  336  269  223  194  160 | 13  6  5  4  3.5  3 | 14  66  5  2  1  12 |
| *Giardia lamblia* |  | - | - |  |
| *Thermus thermophilus* | 1 | 439 | 8 | 100 |
| Horse spleen ferritin type I ^*^ | 3 | *1320*  *880*  *440* | - | 16  16  68 |
| Bovine serum albumin ^*^ | 4 | 264  198  132  66 | - | 7  45  19  29 |

^*^ Taken from ^1^

**Supplementary Table 3.** Properties of the aggregates of TIM observed using CN-PAGE

(HEPES 133.2 mM imidazole 50 mM)

| **Organism or protein** | **Number of aggregates** | **Estimated molecular mass of the aggregates** | **Number of dimers in the aggregate** | **Relative distribution**  **(%)** |
| --- | --- | --- | --- | --- |
| *Trypanosoma cruzi* | 3 | - | - | 11  17  72 |
| *Trypanosoma brucei* | 3 | - | - | 3  2  95 |
| *Entamoeba histolytica* | 6 | 932  532  354  228  76  42 | 17  10  6.5  4  1.5  1 | 53  6  2  17  7  15 |
| *Homo sapiens* | 2 | 1039  31 | 19  0.5 | 76  24 |
| *Homo sapiens 6H* | 2 | 1034  36 | 19  0.5 | 84  16 |
| *Leishmania mexicana* | 4 | - | - | 9  15  12  64 |
| *Plasmodium falciparum* | 3 | 590  354  40 | 11  6.5  1 | 55  7  38 |
| *Taenia solium* | 3 | 651  487  33 | 12  9  0.5 | 60  29  11 |
| *Saccharomyces cerevisiae* | 5 | 1166  537  308  217  43 | 21  10  5.5  4  1 | 5  51  2  13  29 |
| *Giardia lamblia* |  | - | - |  |
| *Thermus thermophilus* | 2 | 587  32 | 11  0.5 | 85  15 |
| Horse spleen ferritin type I ^*^ | 3 | *1320*  *880*  *440* | - | 6  19  75 |
| Bovine serum albumin ^*^ | 4 | 264  198  132  66 | - | 14  43  37  6 |

^*^ Taken from ^1^


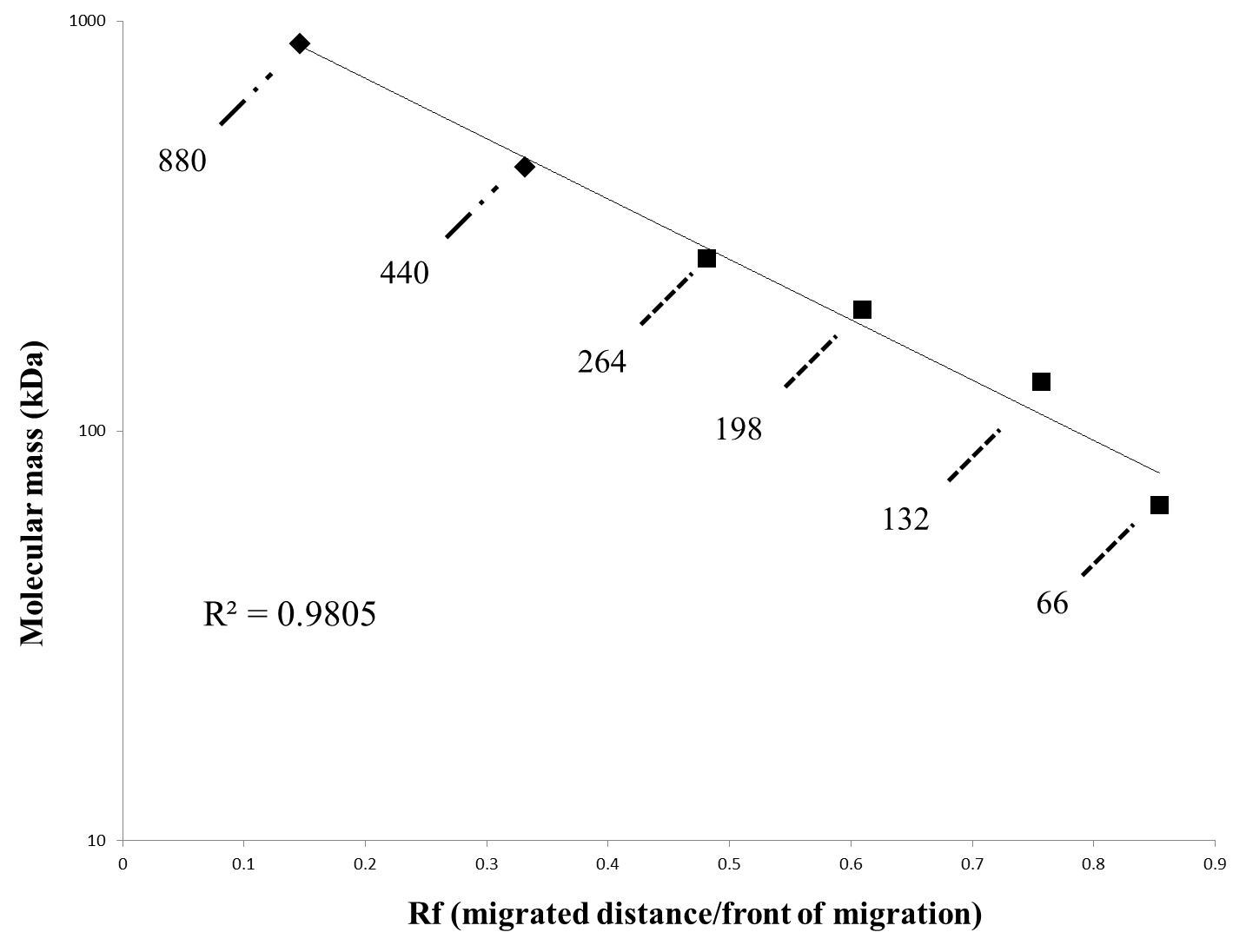


**Figure S2.** **Linear regression plot used to calculate the molecular mass of the aggregates of TIM observed using CN-PAGE with HEPES 40.0 mM and imidazole 15.3 mM.** The logarithms of the molecular masses of the well characterized oligomers of horse spleen ferritin type I (◆) and bovine serum albumin (◼) were plotted against their relative migrated distance (Rf).


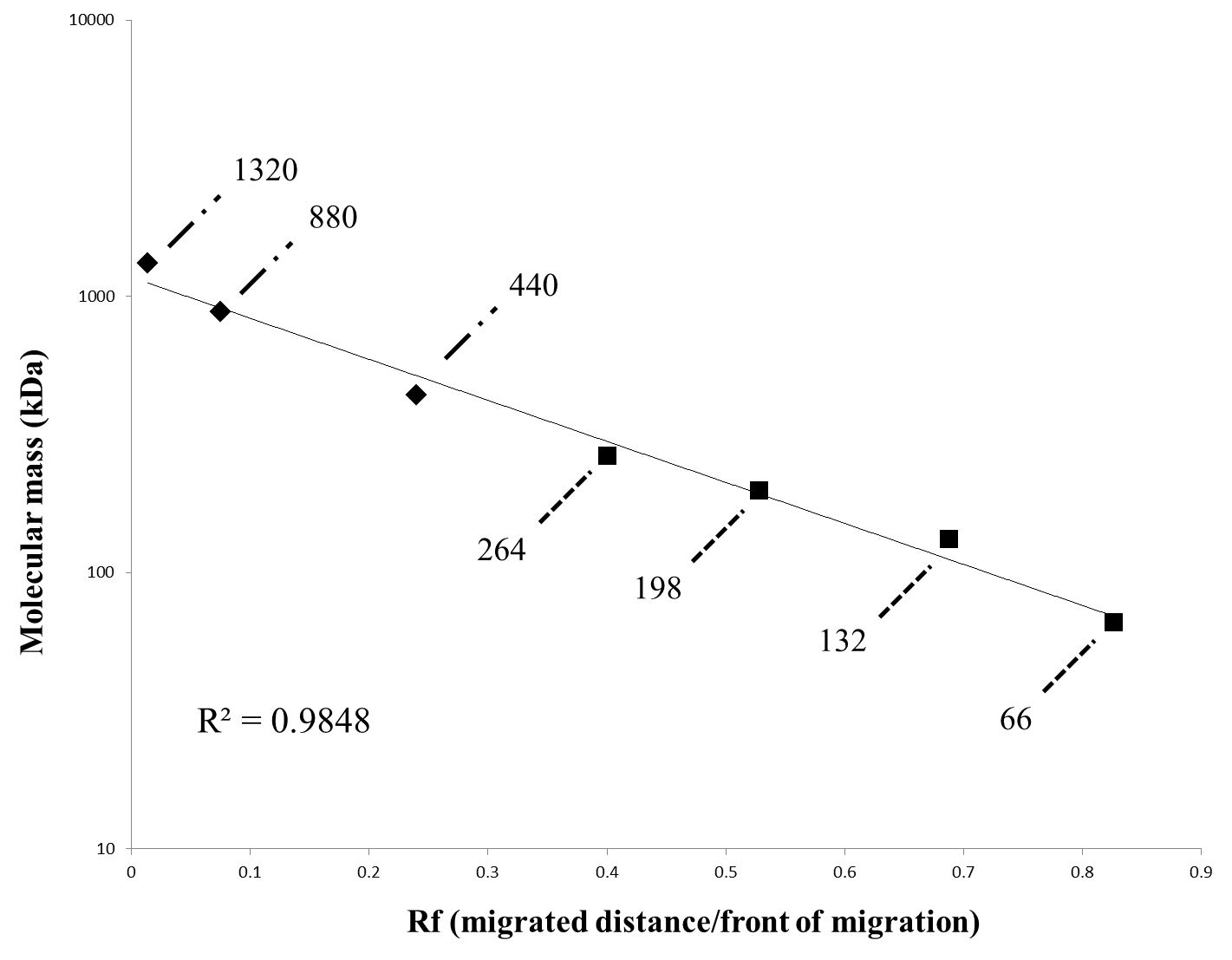


**Figure S3.** **Linear regression plot used to calculate the molecular mass of the aggregates of TIM observed using CN-PAGE with HEPES 88.9 mM and imidazole 34.0 mM).** The logarithms of the molecular masses of the well characterized oligomers of horse spleen ferritin type I (◆) and bovine serum albumin (◼) were plotted against their relative migrated distance (Rf).


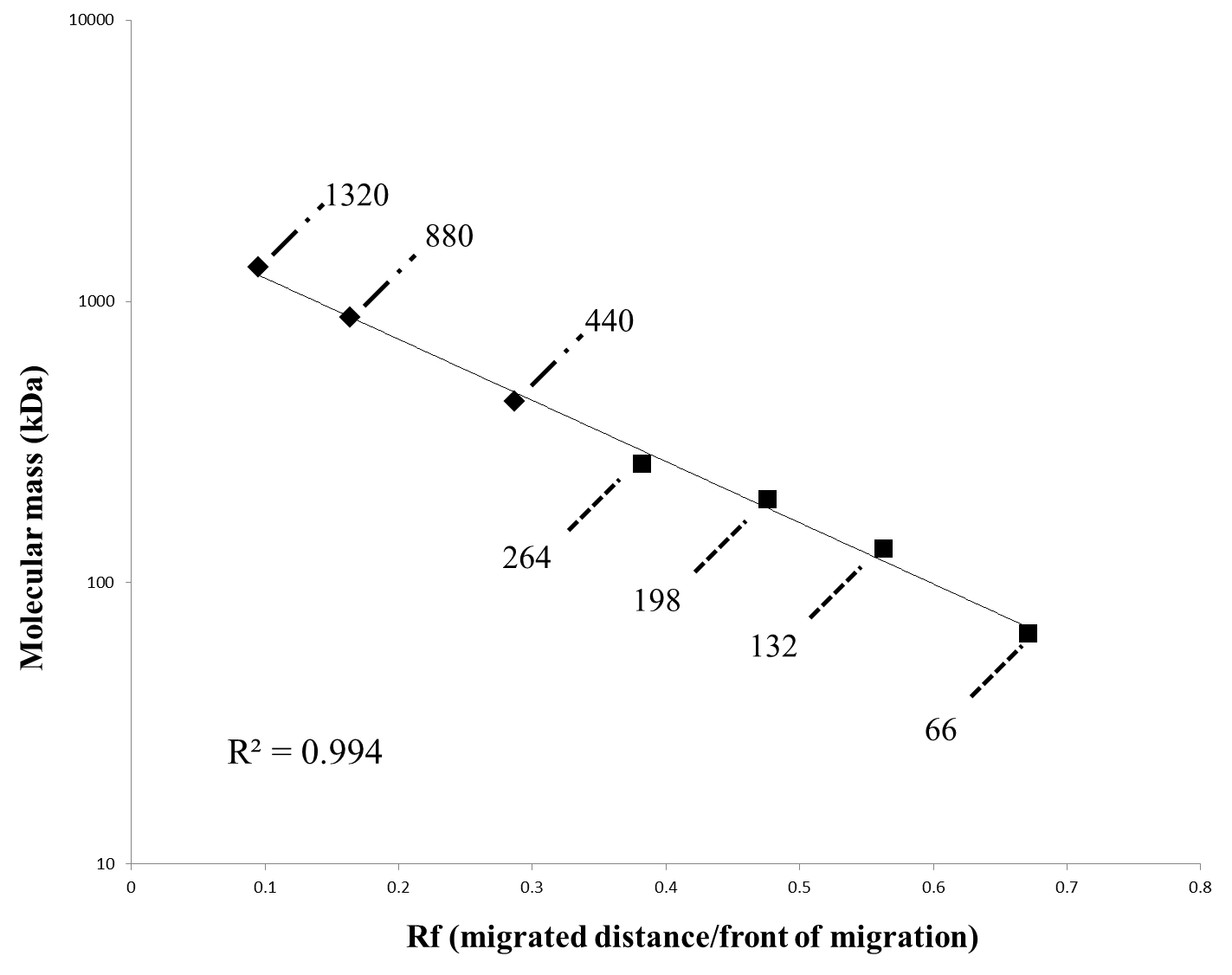


**Figure S4.** **Linear regression plot used to calculate the molecular mass of the aggregates of TIM observed using CN-PAGE with HEPES 133.2 mM and imidazole 50.0 mM.** The logarithms of the molecular masses of the well characterized oligomers of horse spleen ferritin type I (◆) and bovine serum albumin (◼) were plotted against their relative migrated distance (Rf).


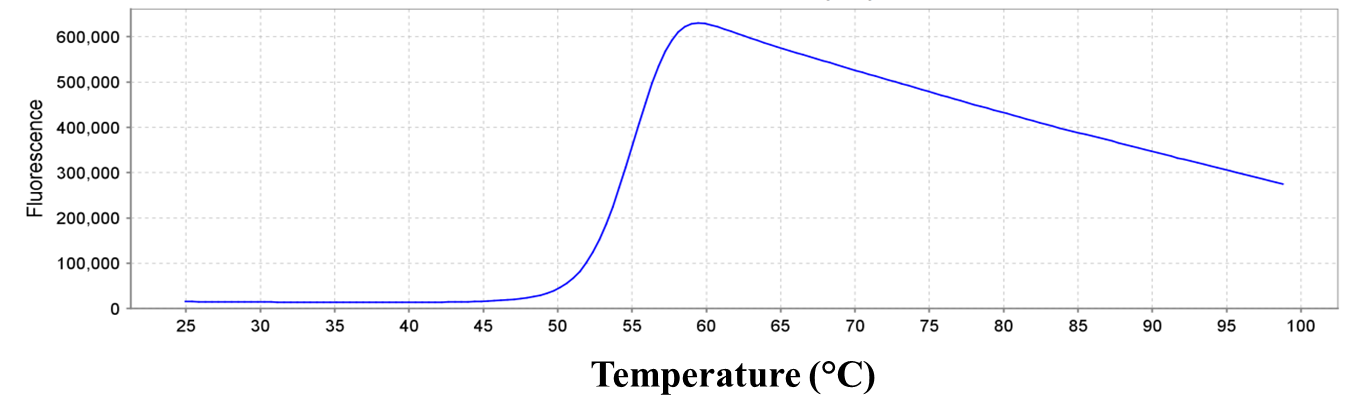


**Figure S5. Determination of the melting temperature of aggregates of TbTIM by thermal shift assay.** The unfolding of native aggregates was followed by the change in the fluorescence of the bound dye SYPRO orange. A temperature gradient (25-99 °C) was applied to 8 µg of a purified fraction with aggregates (those shown with the purple arrow head in Figure 6A, were used).


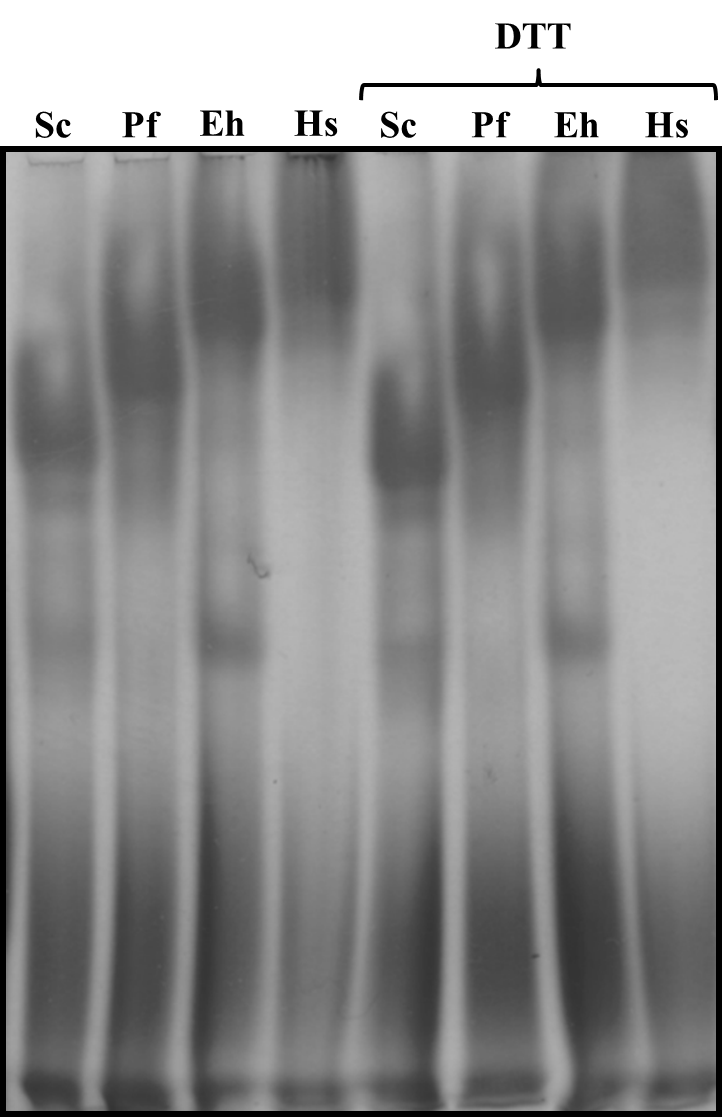


**Figure S6. Effect of the reducing agent dithiothreitol (DTT) on aggregates of native TIM from several species.** Purified ScTIM (Sc), PfTIM (Pf), EhTIM (Eh) and HsTIM (Hs) were incubated 30 min at 25 °C in presence of 10 mM of DTT and analysed by CNE.


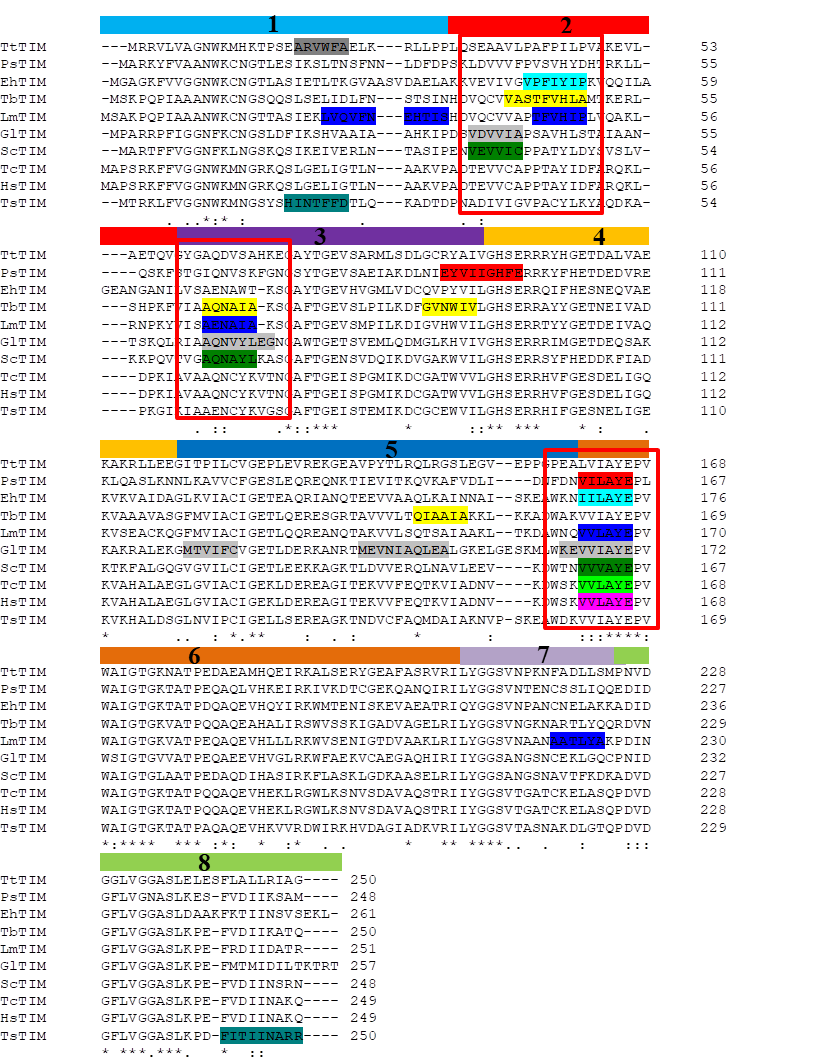


**Figure S7.** **Amylogenic regions predicted for the TIMs from ten different species.** Sequence alignment of TIM from ten different species. The highlighted regions in different colors in the sequences represent a predicted amylogenic region. The color bars above the sequences indicate the regions of the TIM described in ^2^. The red boxes indicate a conserved amylogenic region.


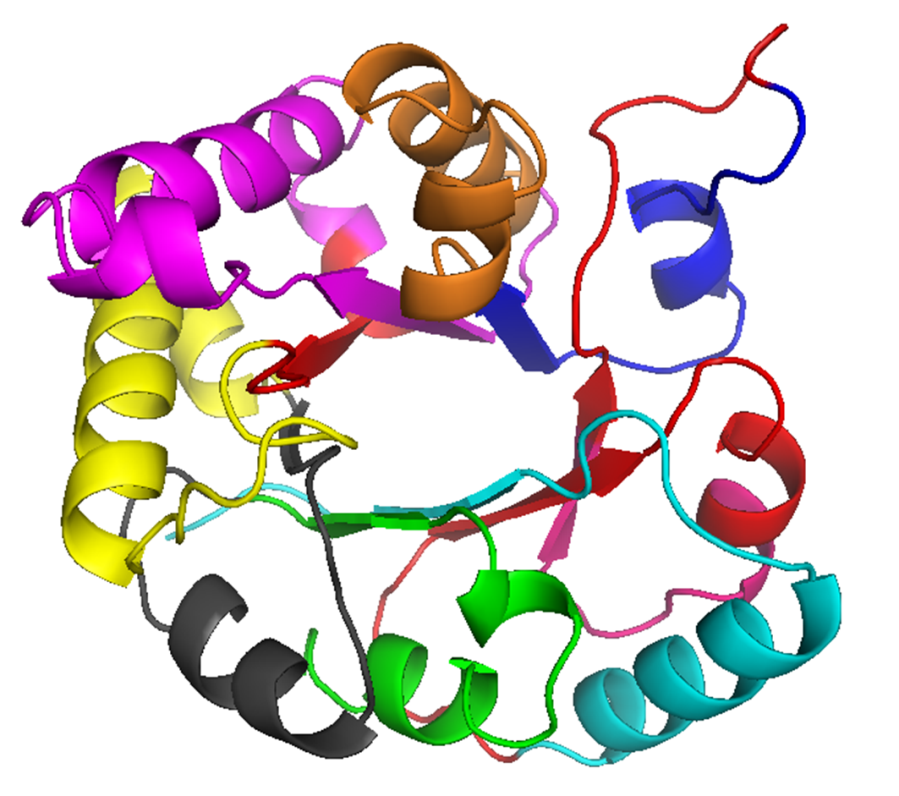


**Figure S8.** **Three-dimensional structure of TIM showing the conserved amylogenic regions.** The structure of TbTIM (PDB 5TIM ^3^) was divided into 8 region as previously described in ^2^. The predicted conserved amylogenic regions are shown in red. Region 1 is shown in cyan, region 2 is shown in pink, region 3 is shown in blue, region 4 is shown in orange, region 5 is shown in magenta, region 6 is shown in yellow, region 7 is shown in black and region 8 is shown in green.

S**upplementary Table 5.** Sequence similarity of the beta amyloid peptide and fragments of the sequence of different TIMs

| **Species** | **Fragment** | **% similarity** |
| --- | --- | --- |
| GlTIM | 177-216 | 25 |
| LmTIM | 174-213 | 23 |
| EhTIM | 181-220 | 20 |
| TcTIM | 175-214 | 20 |
| HsTIM | 173-212 | 18 |
| ScTIM | 171-210 | 15 |
| TbTIM | 174-213 | 15 |

**Supplementary Table 6.** Percentage of identity between the sequences of the TIMs studied in this work

| **Enzyme** | **TbTIM** | **TcTIM** | **LmTIM** | **HsTIM** | **TsTIM** | **GlTIM** | **ScTIM** | **EhTIM** |
| --- | --- | --- | --- | --- | --- | --- | --- | --- |
| **TbTIM** |  | 73% | 69 % | 50 % | 45 % | 43 % | 56 % | 41 % |
| **TcTIM** | 73 % |  | 68 % | 49 % | 47 % | 43 % | 53 % | 42 % |
| **LmTIM** | 69 % | 68 % |  | 57 % | 56 % | 44 % | 44 % | 57 % |
| **HsTIM** | 50 % | 49 % | 57 % |  | 58 % | 44 % | 52 % | 44 % |
| **TsTIM** | 45 % | 47 % | 56 % | 58 % |  | 46 % | 48 % | 41 % |
| **GlTIM** | 43 % | 43 % | 44% | 44 % | 46 % |  | 47 % | 41 % |
| **ScTIM** | 56 % | 53 % | 44 % | 52 % | 48 % | 47 % |  | 36 % |
| **EhTIM** | 40 % | 42 % | 57 % | 44 % | 41 % | 41 % | 36 % |  |

**References for Supplementary Information**

1. Fiala, G. J., Schamel, W. W. A. & Blumenthal, B. Blue native polyacrylamide gel electrophoresis (BN-PAGE) for analysis of multiprotein complexes from cellular lysates. *J. Vis. Exp.* e2164 (2011). doi:10.3791/2164

2. Rodríguez-Bolaños, M., Cabrera, N. & Perez-Montfort, R. Identification of the critical residues responsible for differential reactivation of the triosephosphate isomerases of two trypanosomes. *Open Biol.* **6**, 160161 (2016).

3. Wierenga, R. K., Noble, M. E. M., Vriend, G., Nauche, S. & Hol, W. G. J. Refined 1.83 Å structure of trypanosomal triosephosphate isomerase crystallized in the presence of 2.4 m-ammonium sulphate. A comparison with the structure of the trypanosomal triosephosphate isomerase-glycerol-3-phosphate complex. *J. Mol. Biol.* **220**, 995–1015 (1991).
